# Neural correlates of attention bias to masked facial threat cues: Examining children at-risk for social anxiety disorder

**DOI:** 10.1016/j.nicl.2018.04.003

**Published:** 2018-04-12

**Authors:** Eran S. Auday, Bradley C. Taber-Thomas, Koraly E. Pérez-Edgar

**Affiliations:** a The Pennsylvania State University, United States; b University at Buffalo, The State University of New York, United States

## Abstract

**Background:**

Behavioral inhibition (BI) is an early-appearing temperament trait and a robust predictor of social anxiety disorder (SAD). Both BI and anxiety may have distinct patterns of emotion processing marked by heightened neural responses to threat cues. BI and anxious children display similar frontolimbic patterns when completing an emotion-face attention bias task with supraliminal presentation. Anxious children also show a distinct neural response to the same task with subliminal face presentations, probing stimulus-driven attention networks. We do not have parallel data available for BI children, limiting our understanding of underlying affective mechanisms potentially linking early BI to the later emergence of anxiety.

**Method:**

We examined the neural response to subliminal threat presentation during an emotion-face masked dot-probe task in children oversampled for BI (*N* = 67; 30 BI, 9–12 yrs).

**Results:**

Non-BI children displayed greater activation versus BI children in several regions in response to threat faces versus neutral faces, including striatum, prefrontal and temporal lobes. When comparing congruent and incongruent trials, which require attention disengagement, BI children showed greater activation than non-BI children in the cerebellum, which is implicated in rapidly coordinating information processing, aversive conditioning, and learning the precise timing of anticipatory responses.

**Conclusions:**

Non-BI children may more readily engage rapid coordinated frontolimbic circuitry to salient stimuli, whereas BI children may preferentially engage subcortical circuitry, in response to limbic “alarms” triggered by subliminal threat cues. These data help reveal the extent to which temperamental risk shares similar neurocircuitry previously documented in anxious adolescents and young adults in response to masked threat.

## Highlights

• All children displayed amygdala activation in response to brief threat cues.
• Non-BI children displayed activation in striatum, PFC and temporal lobes.
• BI children showed greater activation in the cerebellum.

## 1 Introduction

Anxiety is a state of tension, worry, and apprehension regarding uncertain, and potentially negative, future events (Gallo et al., 2012). Although anxiety serves an important evolutionary safety function by increasing vigilance and improving our ability to identify threats, anxiety can be functionally impairing when excessive or activated in the absence of real threat. Anxiety disorders are often early appearing and entrenched, with a lifetime prevalence of 32.4% for children and 33.7% for adults (Kessler et al., 2012), and a lifetime prevalence of 8.6% for children and 13% for adults (Kessler et al., 2012) for social anxiety disorder (SAD).

Behavioral inhibition (BI), a temperamental response to unfamiliar stimuli (Kagan et al., 1984), is a robust predictor of SAD (Chronis-Tuscano et al., 2009; Clauss and Blackford, 2012; Pérez-Edgar and Fox, 2005), and is associated with up to a sevenfold increase in risk (Clauss and Blackford, 2012). BI and anxiety are also linked by studies finding similar neural functioning in anxious children and behaviorally inhibited children (e.g., Guyer et al., 2006; Helfinstein et al., 2012; Pérez-Edgar et al., 2007; Pérez-Edgar et al., 2014) and in adults with a history of behavioral inhibition (Schwartz et al., 2003a, Schwartz et al., 2003b), even before the emergence of clinical anxiety. Recent work has focused on elucidating underlying mechanisms that may account for the neural and behavioral links between BI and anxiety.

For example, vigilance (or attentional bias) toward threat cues may be a causal mechanism in anxiety (Amir et al., 2005; Bar-Haim, 2010; MacLeod et al., 1986; for review see Cisler and Koster, 2010), underpinned by perturbations in the frontolimbic system's response to exposure to threat (for reviews see: Duval et al., 2015; Freitas-Ferrari et al., 2010; Phan, 2015; Taylor and Whalen, 2015). This neural system is sensitive to the duration of threat exposure as the relative weight of reactive (limbic) and regulatory (prefrontal) responses shift with brief (Lipka et al., 2011; for review see Brooks et al., 2012) versus sustained (e.g., Hardee et al., 2013) exposure to threat. Behaviorally inhibited children show attentional bias to sustained threat, which in turn is associated with variations in social behavior and anxiety (Morales et al., 2015; Morales et al., 2016; Pérez-Edgar et al., 2010; Pérez-Edgar et al., 2007, Pérez-Edgar et al., 2011). In addition, behaviorally inhibited children (Fu et al., 2017), young adults with a history of BI (Hardee et al., 2013), and clinically anxious adolescents (Monk et al., 2006) display heightened prefrontal response when completing an attention bias task with a sustained threat presentation. This suggests that attentional bias for threat may be involved in the BI-anxiety link.

However, no studies have examined brain functioning in response to brief threat in behaviorally inhibited children. BI is rooted in heightened reactivity to novel or threatening stimuli, and neural models suggest that, like anxiety (e.g., Davis and Whalen, 2001; Rapee and Heimberg, 1997), BI may be driven by a heightened “reactive” (or stimulus-driven) neural system (Kagan et al., 1987; Fox et al., 2001). Nonetheless, most studies have used sustained (i.e., 500 ms or longer) presentation of threat when examining attention bias to threat which is more sensitive to top-down, rather than stimulus-driven, neural processing (e.g., Britton et al., 2012; Monk et al., 2006; Telzer et al., 2008). In these studies, the data have pointed to a stronger role for frontal functioning (Britton et al., 2012; Monk et al., 2006; Telzer et al., 2008), as opposed to the hypothesized reactive—or limbic—response central to anxiety and BI. One of the few studies using brief (subliminal) threat exposure found greater right amygdala activation in anxious adolescents and a negative amygdala-vlPFC coupling that was weaker in anxious adolescents than in healthy controls (Monk et al., 2008). It is not clear whether children with BI will also show a similar pattern. Detailing patterns of reactive neural functioning in at-risk children *before* the emergence of disorder may help us better understand the attentional and neural mechanisms supporting the interrelation between early temperament and the later emergence of anxiety.

### 1.1 Attentional bias toward threat (ABT) and anxiety

Attentional biases toward threat-relevant lexical and pictorial threat cues have been observed across anxiety disorders (reviews by Mogg and Bradley, 1998; Williams et al., 1997) for both children and adults (Bar-Haim et al., 2007; Dudeney et al., 2015). Moreover, evidence of a brief (and arguably automatic) processing bias for threatening information in both clinical and non-clinical anxiety can be inferred from vigilant reactions to masked threat cues (presented outside awareness) in visual probe (Bradley et al., 1997; Mathews and MacLeod, 1985; Mogg and Bradley, 2002) and visual search (Byrne and Eysenck, 1995; Gilboa-Schechtman et al., 1999) paradigms.

The current study used the dot-probe task (Bar-Haim et al., 2007) to explore children's responses to briefly presented threat cues. The dot-probe task is widely used to study responses of children and adolescents to facial threat cues (i.e., angry faces; e.g., Britton et al., 2012; Monk et al., 2006, Monk et al., 2008; Price et al., 2014; Telzer et al., 2008). In this task, each trial presents a pair of faces (either neutral-neutral or neutral-threat) followed by a probe replacing one of the faces. Biased attention toward threat is quantified in the neutral-threat trials by slower reaction times to the probes that replace the neutral face (incongruent trials) compared to the probes that replace the threat face (congruent trials). In line with the neural parallels between anxiety and BI, children (Broeren et al., 2011; Pérez-Edgar et al., 2011; White et al., 2017a) and adolescents (Pérez-Edgar et al., 2010) with stable childhood BI who also display heightened ABT present increased levels of social withdrawal and socially anxious behavior, even in the absence of group level differences in ABT.

### 1.2 Neural underpinnings for attention to threat cues

Functional neuroimaging studies report amygdala hyperactivation in response to threat cues, particularly when social, in individuals with SAD compared to healthy controls (Taylor and Whalen, 2015). Furthermore, symptom severity in SAD is positively correlated with amygdala activation (Duval et al., 2015). Specifically, studies have implicated the amygdala in supporting vigilance through *immediate* threat cue processing (Davis and Whalen, 2001; Phillips et al., 2003a, Phillips et al., 2003b). This initial threat processing precedes subsequent higher-level emotion cue processing (i.e., emotion regulation or modulation; Hariri et al., 2003; Nomura et al., 2004; Ochsner and Gross, 2005).

In addition to the amygdala, the insula is also implicated in the etiology of anxiety disorders (e.g., Damsa et al., 2009; Paulus and Stein, 2006) as it responds in anticipation of uncertainty, facilitates the processing of salient, goal-relevant information, and, through connections with other brain regions (e.g., hypothalamus, amygdala, mPFC), regulates the autonomic nervous system (Menon and Uddin, 2010; Taylor and Whalen, 2015). Hyperactive insular responses to social threat cues (Shah et al., 2009) and emotional faces (Klumpp et al., 2013) have also been noted in SAD. Finally, the distributed neural network underlying SAD also encompasses the striatum and dorsal ACC (dACC; Amir et al., 2005; Freitas-Ferrari et al., 2010; Goldin et al., 2009), indicating striatal hypoactivity and dACC hyperactivity.

Studies have also examined patterns of response to threat exposure during the dot-probe task in pediatric anxiety (Britton et al., 2012; Monk et al., 2006, Monk et al., 2008; Price et al., 2014; Telzer et al., 2008), almost exclusively under prolonged exposure durations (e.g., 500 ms). Monk et al. (2006) reported that youth with GAD, compared to healthy controls, exhibited greater right ventrolateral PFC (vlPFC) activation to 500 ms presentations of angry faces. No group differences were reported in amygdala activation (Monk et al., 2006). Price et al. (2014) reported that non-anxious youth showed deactivation between rostrodorsal ACC (rdACC) and limbic regions (parahippocampal/hippocampal) during incongruent trials (when attention was deployed away from threat). Anxious youth, in contrast, displayed rdACC deactivation and attenuated deactivation of limbic regions. Telzer et al. (2008) reported that trait anxiety was positively associated with right dorsolateral PFC (dlPFC) activation in an incongruent-congruent trials contrast. Finally, Britton et al. (2013) reported greater negative activation in vlPFC during a magnetoencephalography (MEG) study in youth with GAD, compared to healthy controls. Together, these studies indicate a cortical response to threat cues in addition to limbic responses that are specific to attention shifting rather than processing threat cues in general.

Similar responses to threat are evident in behaviorally inhibited adolescents and young adults (Hardee et al., 2013; Fu et al., 2017; Schwartz et al., 2003a, Schwartz et al., 2003b; White et al., 2010). Hardee et al. (2013) showed that behaviorally inhibited individuals exhibit greater threat-related amygdala-PFC connectivity, specifically dlPFC and anterior insula. Fu et al. (2017) reported increased right dlPFC activation in behaviorally inhibited children during the dot-probe task at 500 ms presentation. Activity in these networks was also associated with internalizing symptoms and anxiety symptoms, respectively. Studies of intrinsic brain networks in behaviorally inhibited children (Roy et al., 2014; Taber-Thomas et al., 2016) and adults (Blackford et al., 2014) have also pointed toward altered functioning in salience (insula, dACC), executive (dlPFC), and frontolimbic (vmPFC) networks. Together, these studies indicate that at-risk individuals show neural activation patterns and circuitry that are similar to those of anxious individuals.

### 1.3 Duration of exposure to threat stimuli

Although almost all of the ABT literature has focused on 500 ms exposures, behavioral and neuroimaging work suggests that patterns of attentional processing shift with varying duration of exposure to threat stimuli. Using eye movement as an index of selective attention, Gamble and Rapee (2009) reported that anxious youth showed a bias in initial orienting away from negative faces (including anger and fear) at 500 ms presentation duration but no between-group differences with prolonged exposure (3000 ms). Another study (Price et al., 2014) reported that anxious youth showed a more vigilant behavioral pattern than control participants during short (200 ms) compared to long (2000 ms) cue presentations and a more avoidant pattern during long presentations.

Prolonged (500 ms) exposure elicits relatively less limbic response and a greater role for frontal regions (Monk et al., 2006), presumably reflecting their regulatory roles (for reviews see: Duval et al., 2015; Taylor and Whalen, 2015). In contrast, in the only study to examine brief (17 ms) threat presentation in clinically anxious adolescents, exposure to angry faces during the dot-probe task was marked by heightened amygdalar arousal, which was positively correlated with anxiety symptom severity (Monk et al., 2008). This study also reported that anxious youth exhibited reduced negative amygdala-vlPFC coupling suggesting that anxious youth could have impaired amygdala modulation by frontal regions.

### 1.4 The current study

Although there is theoretical (e.g., Kagan, 2001; Kagan and Fox, 2006) and empirical (Fox et al., 2001; Schwartz et al., 2003a, Schwartz et al., 2003b; Roy et al., 2014) support for the hypothesis that BI and risk for anxiety are both marked by heightened reactive neural responses to threat in stimulus-driven attention networks, there is little direct evidence in support of that notion. No study to date has explored the response to subliminal threat presentation in BI. Using extremely brief or masked presentations of threat may better clarify the nature of amygdala–prefrontal (especially amygdala-vlPFC) involvement in the development of anxiety. These data could reveal the extent to which temperamental risk encompasses the full neurocircuitry of the threat response previously documented in anxious adolescents and young adults. This information would further enhance our understanding of the mechanisms that may convert risk to disorder and refine our targets of prevention and intervention.

The present study examined attentional bias toward threat in children ages 9–12 years characterized for BI via parental report. Based on the available literature (Monk et al., 2008), we hypothesized that behaviorally inhibited children, relative to non-inhibited children, will show greater amygdala activation to briefly presented angry face-stimuli. Extrapolating from the literature, we further hypothesized that behaviorally inhibited children, relative to non-inhibited children, will show greater activation in the insula and dACC, as these regions are conceptualized to be part of the salience network (Taber-Thomas et al., 2016), potentially alerting children to threat, and reduced striatal activation in response to briefly presented angry face-stimuli.

## 2 Method

### 2.1 Participants

Participants were one-hundred twelve 9–12-year-olds (M = 11.00, SD = 1.00; 63 female), drawn from a larger, multi-visit study of AB and temperamental risk for anxiety. Families were recruited through a university database of families interested in participating in research studies, community outreach, and word-of-mouth. Participants were screened based on parent-report using the Behavioral Inhibition Questionnaire (BIQ; Bishop et al., 2003). Cut-off scores were based on previous studies of extreme temperament in children ages 4 to 15 years (Broeren and Muris, 2010) and our initial screening of 705 children to establish the expected BI distribution (Liu et al., 2018). For the screening sample, scores ranged from 30 to 182 (Mean = 90.4, SD = 30.7), which is in line with published data (Broeren and Muris, 2010; White et al., 2016). Our cut-off criteria identified children with extreme (top 25%) BI scores. Fifty-five children meeting BI criteria (i.e., scored high on both the social novelty subscale and grand total score (*N* = 29), the social novelty subscale only (≥60; *N* = 23), or the grand total score only (≥119; *N* = 3)) participated in the fMRI session. Fifty-seven children participating as sex- and age-matched non-BI control participants in the larger study were recruited for the fMRI sub-component (Table 1). BIQ scores for the included participants ranged from 41 to 165. Data presented here are from the BI children's first (baseline) visit.

**Table 1:** Demographic characteristics and descriptive statistics (mean and standard deviation).

	Included Participants			Excluded Participants	
	Full Sample	BI	Non-BI	BI	Non-BI
Demographics					
N Gender (M/F)	67 29/38	30 15/15	37 14/23	25 9/16	20 11/9
Age	10.98 (1.00)	10.73 (0.97)	11.17 (1.00)	10.88 (0.96)	11.26 (1.03)
IQ	112.35 (13.27)	111.59 (10.42)	112.97 (15.58)	107.96 (14.74)	106.35 (11.82)
BI Characterization					
Total BIQ	95.44 (29.57)	121.03⁎⁎⁎ (18.05)	74.69⁎⁎⁎ (18.78)	129.38⁎⁎⁎ (20.71)	70.30⁎⁎⁎ (23.79)
Number of children BIQ cut-off (≥119) only	NA	1	NA	2	NA
Number of children novelty cut-off (≥60) only	NA	16	NA	7	NA
Symptom Characterization					
Total SCARED	10.28 (7.90)	13.79⁎⁎ (7.09)	7.00⁎⁎ (7.26)	18.69⁎⁎ (12.95)	7.13⁎⁎ (5.22)
Social Anxiety Symptom Levels (%)	12.31 (22.25)	22.25 (26.17)	4.78 (15.20)	33.78 (32.03)	3.85 (12.85)
Dot-Probe Task Performance					
Valid Dot-Probe Trials (%)	90.71 (0.07)	90.56⁎ (6.63)	90.84⁎⁎ (7.65)	83.66⁎ (15.77)	81.03⁎⁎ (19.42)
RTs for Valid Dot-Probe Trials (ms)	606.88 (79.94)	597.93 (81.79)	613.89 (78.86)	608.61 (79.68)	598.35 (70.36)
AB Score	−3.91 (17.59)	−2.68 (16.48)	−4.88 (18.58)	−7.24 (21.46)	−14.65 (47.53)

Raw values are presented to assist interpretation. Among both included and excluded participants, behaviorally inhibited children scored higher on total BIQ and total SCARED than non-inhibited children. BIQ = Behavioral Inhibition Questionnaire; SCARED = Screen for Child Anxiety Related Emotional Disorders; AB = attention bias.

⁎ *p* *<* .05 for comparisons of BI to Non-BI.

⁎⁎ *p* < .01 for comparisons of BI to Non-BI.

⁎⁎⁎ *p* < .001 for comparisons of BI to Non-BI.

While mindful of data loss, we chose a conservative approach to data inspection due to recent concern regarding pediatric neuroimaging (Greene et al., 2016; Leroux et al., 2013; Raschle et al., 2012). Therefore, data were carefully inspected for quality assurance, with participants removed from analyses for meeting one of the exclusion criteria: exceeding movement thresholds (>3 mm, N = 23), poor task performance (>25% of trials with missing responses, inaccurate responses or outlier RTs, *N* = 1), or technical problems with the triggering system (N = 2). Three participants were excluded due to being a member of a sibling pair that also participated in the study (in two sibling pairs one sibling was BI and the other was non-BI, therefore, the non-BI sibling was excluded; we chose randomly for the sibling pair that were both BI). We excluded siblings to preclude any possible effect of shared environmental/genetic background on the measured brain activity during task presentation (Gregory and Eley, 2007).

Finally, participants were excluded if there was a complete absence of visual cortex activation when task stimuli were present versus absent (*N* = 16). We initially examined the uncorrected threshold using the SPM default of *p* = .001. We then examined clusters that survived the FWE-correction and FDR-correction. We excluded participants who had clusters in the visual cortex that survived neither the uncorrected nor corrected thresholds. This method is common practice when utilizing visual tasks in fMRI studies, based on the argument that lack of activation in the occipital cortex in a task-versus-baseline contrast suggest lack of attention to the task. These data should be excluded from the analyses as they do not reflect the intended manipulation (e.g., Ganis et al., 2004; Kosslyn and Thompson, 2003). In our study, our quality assurance contrast was of task (brief-face presentation and then a mask, presented for a total of 500 ms) versus baseline, which should elicit activation in visual cortex (regardless of duration of face stimuli presentation).

The final sample consists of 67 children (*M*_*age*_ = 10.97 years; *SD* = 1.00; 38 females; 30 BI). Included and excluded participants did not differ in age, gender, IQ, BIQ scores, AB Score, or anxiety symptoms (*p*'s > .08, Table 1). Within the final sample, the anxiety measure was missing for 7 participants (all non-BI). They did not differ from the sample in age, gender, IQ, total BIQ scores, or AB score (*p*'s > .52). Forty-two participants also provided data for the unmasked (500 ms) version of the task for Fu et al. (2017).

The study was approved by the institutional review board at the Pennsylvania State University. Parents and children provided written consent/assent.

### 2.2 Measures

***Behavioral inhibition*** was assessed using the BIQ (Bishop et al., 2003), a 30-item instrument that measures the frequency of BI-linked behavior in the domains of social and situational novelty (plus a summed total score) on a seven-point scale ranging from 1 (“hardly ever”) to 7 (“almost always”). Questions were edited to be more appropriate for the target age range in the current study (e.g. reference to preschool, kindergarten, and childcare was removed for the question: “Happily separates from parent(s) when left in new situations for the first time (e.g. kindergarten, preschool, childcare)”). The questionnaire has adequate internal consistency, construct validity, and validity in differentiating inhibited from non-inhibited children (Bishop et al., 2003). Parent report with the BIQ correlates with laboratory observations of BI in social contexts (Dyson et al., 2011), and maternal report of BI is predictive of anxiety level over time (Chronis-Tuscano et al., 2009). The BIQ had good internal consistency in the present study (Cronbach's α = 0.91).

***Anxiety symptoms*** were assessed using two measures: the parent-report version the Screen for Child Anxiety Related Emotional Disorders (SCARED, Birmaher et al., 1999) and the parent-report on the computerized Diagnostic Interview Schedule for Children (C-DISC).

The SCARED is a 41-item instrument assessing symptoms of panic disorder, generalized anxiety, separation anxiety, social phobia, and school phobia defined in the Diagnostic and Statistical Manual of Mental Disorders (DSM-IV, 1994). Parents rated the frequency with which their children experience each symptom on three-point scales (0 = “almost never”, 1 = “sometimes”, and 2 = “often”). Subscale scores were summed to create the total score. The SCARED has satisfactory psychometric properties in both clinical (Birmaher et al., 1999) and community samples (Hale et al., 2005), and it offers a valuable tool to predict specific anxiety disorders in clinically-referred youths (Muris et al., 2004). It had good internal consistency in the present study (Cronbach's α = 0.90).

The C-DISC is an interviewer administered version of the computerized Diagnostic Interview Schedule for Children used to assess for anxiety symptoms and disorders. The C-DISC is a comprehensive, structured interview that covers 36 mental health disorders for children & adolescents, using DSM-IV criteria. Most questions are worded so that they can be answered “yes”, “no”, and “somewhat” or “sometimes”. Questions reference the 4 weeks, 6 months, and 1 year prior to the interview. The interviews were administered by trained research assistants with the primary caregiver and scored using the software's algorithm.

**Fig. 1:**
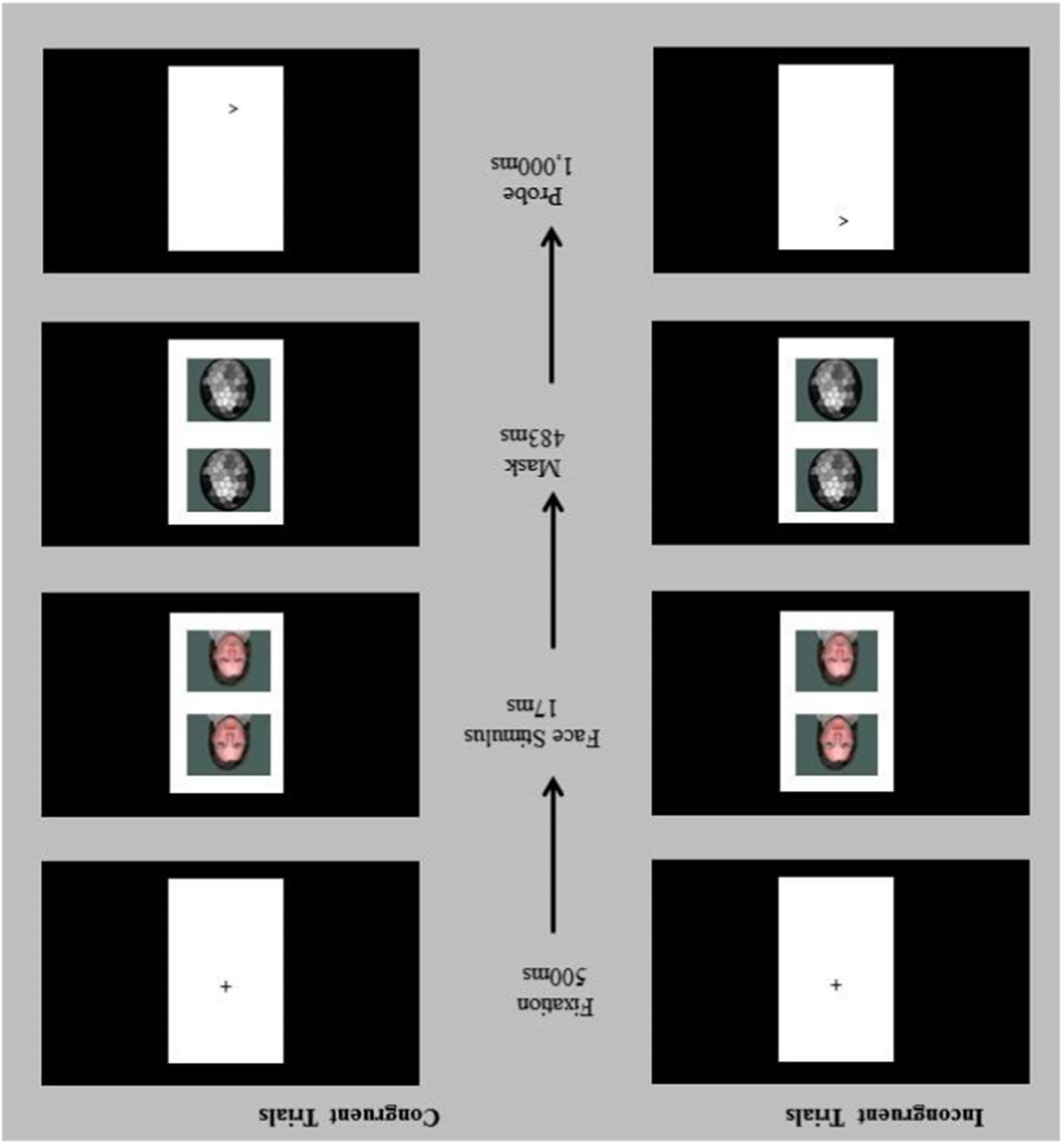
Schematic of the masked dot-probe task illustrating congruent and incongruent trials. In incongruent trials, the probe appeared on the opposite side of the angry face. In congruent trials, the probe appeared on the same side as the angry face. The same actor appeared for both expressions within a trial. Each probe direction (<left or >right) appeared for half of the trials.

***ABT*** was assessed in an event-related fMRI dot-probe task modeled on previous studies in anxious (Monk et al., 2006, Monk et al., 2008) and BI (Hardee et al., 2013) youth, implementing the TAU-NIMH Toolbox (Abend et al., 2014). Stimuli were displayed on a projector with resolution of 1024(H) by 768(V) at 75 Hz. At the viewing distance of 143 cm, the display area was 20°(H) ∗ 16°(V). The visual angles for the face image are 1.80°(H) ∗ 1.36°(V). Each trial (2500 ms) began with a 500 ms fixation cross, followed by a face pair displayed on the top and bottom of the fixation point for 500 ms (17 ms of face display and the remaining time masked; Fig. 1). The faces and fixation point were replaced by an arrow-probe presented for 1000 ms in the location of one of the preceding faces. Participants indicated whether the arrow pointed to left or right by pressing a button (response recorded for 2500 ms). The inter-trial interval varied between 250 ms to 750 ms (average 500 ms).

The task displayed face pairs of 20 actors (NimStim; Tottenham et al., 2009) across 320 trials divided into two task runs. There were 80 trials for each of 3 trial types in each run: 1) congruent trials in which the angry-neutral face pair was followed by an arrow in the same position as the angry face; 2) incongruent trials in which the angry-neutral face pair was replaced by the probe appeared on the opposite side of the angry face; and 3) neutral-neutral trials with the probe presented on either location. Eighty blank trials were added to each run to create jitter and serve as an additional baseline. Angry-face location, arrow-probe location, arrow-probe direction, and actors were counterbalanced for each participant.

Prior to fMRI acquisition, participants practiced the dot-probe task in a mock scanner. Participants repeated the 10 practice trials until achieving at least 80% accuracy. The mock scanner procedures were added to familiarize children with the fMRI environment, sounds, and task procedures, and train them to be as still as possible during scans. During mock and actual data acquisition, task stimuli were viewed with mirrors on the head coil. Padding was used to limit head and body movement.

### 2.3 fMRI data acquisition

Imaging data were acquired in two 170 volume runs (180 task trials/run) on a 3-Telsa MRI scanner (MAGNETOM Trio with Tim system, Siemens Medical Solutions, Erlangen, Germany), with descending acquisition of 43 continuous 3 mm axial slices angled approximately 15° above the AC-PC line. T2*-weighted echo-planar sequence was applied with a repetition time (TR) of 2500 ms, echo time (TE) of 25 ms, flip angle of 80°, and field of view (FoV) of 192 mm. The voxel size was 3 × 3 × 3 mm, and the image matrix was 64 × 64. High-resolution T1-weighted structural scans were also acquired using a magnetization prepared gradient echo sequence (MP-RAGE) (176 1 mm slices, TR = 1700, TE = 2.01, flip angle = 9°, FoV = 256 mm, voxel size = 1 × 1 × 1 mm; 256 × 256 matrix, T1 = 850 ms).

### 2.4 fMRI data preprocessing

Data were preprocessed and analyzed using SPM8 (Wellcome Trust Center for Neuroimaging, London, UK) and MATLAB (Version 7.14.0; Mathworks, Inc., Natick, MA). Functional images were realigned to the first image of each run. The T1 was coregistered to the mean realigned functional image, and then normalized to the Cincinnati Children's Hospital Pediatric Brain Template for 9–12 year olds (Wilke et al., 2002) using SPM's unified segmentation. These normalization parameters were then applied to the functional time series, which were then spatially smoothed with a 6 mm kernel.

### 2.5 Data analysis

#### 2.5.1 Behavioral data

We excluded trials with missing responses, incorrect responses, RTs outside a 150–2000 millisecond window post-probe presentation, and RTs ±2 standard deviations of the individual child's mean (derived from included trials). The children completed the task with relative ease, with roughly 90% valid trials, and only one child excluded for poor task performance. When examining specific RT-linked patterns of data loss, we excluded a maximum of 3 trials for being too fast and no trials were excluded for being too slow. Trials were excluded from 5 of the 67 participants.

AB scores for each participant were calculated as mean RT to the probes on the incongruent trials minus mean RT to the probes on congruent trials.

#### 2.5.2 fMRI data

At the first-level, fixed-effects analysis was conducted for each participant with four regressors created for the trial types: neutral-neutral, congruent, incongruent, and invalid (missing responses, incorrect responses and responses with outlier RTs), along with motion regressors specified based on the 24-parameter autoregressive model (Friston et al., 1996). Task-related regressors were modeled by convolving event onset times with a canonical hemodynamic response function. The t-contrasts estimated for examining signal change corresponding to threat-related AB were threat-neutral trials versus neutral-neutral trials (Threat > Neutral) and incongruent-versus-congruent in the threat-neutral trials (Incongruent > Congruent).

The contrast images containing parameter estimates for individual participants were entered into two second-level, random-effects analyses: (1) whole-sample Threat > Neutral: to examine regions activated in the Threat > Neutral contrast across all participants in a one-sample *t*-test, and (2) between-group Threat > Neutral: to compare Threat > Neutral activation between the BI and non-BI groups in a two-sample *t*-test. In addition to SPM's implicit masking procedure set to include voxels with at least 20% of mean signal, an explicit brain mask was applied to limit the analyses to voxels with >20% probability of being gray matter.

To correct for multiple comparisons at the whole-brain cluster-wise alpha of 0.05 with a voxel-wise p-threshold of 0.005 (based on the average smoothness of our data: FWHM values of 11.3 mm ∗ 11.5 mm ∗ 9.9 mm in x, y, and z dimensions), 10,000 Monte Carlo simulations using a 2 × 2 × 2 voxel mask in AFNI revised 3dClustSim program (May 2015) found that a minimum cluster size of 86 contiguous voxels was necessary to achieve significance. Following prior literature (e.g. Hardee et al., 2013; Monk et al., 2006, Monk et al., 2008), small-volume correction (SVC) was applied based on a priori anatomical region of interest (ROI) to assess clusters of activation within the amygdala. Left (79 voxels) and right (87 voxels) amygdala were defined using the AAL Atlas (Tzourio-Mazoyer et al., 2002) provided in the WFU Pick Atlas (Maldjian et al., 2003). Monte Carlo simulations (smoothness of 10.4 × 7.4 × 9.6 mm FWHM) determined that a minimum 13-voxel cluster size for both left and right amygdala was needed to achieve a cluster-wise *p* = .05 at voxel-wise *p* = .005.

## 3 Results

### 3.1 Behavioral results

One-sample *t*-tests examining AB to angry faces were non-significant for the whole sample, *t*(66) = −1.80, *p* = .07, *d* = −0.44, as well as separately for BI, *t*(29) = −0.87, *p* = .39, *d* = −0.32, and non-BI groups, *t*(36) = −1.60, *p* = .12, *d* = −0.53. The BI vs. non-BI group comparison of AB was not significant, *t*(65) = −0.50, *p* = .62, *d* = −0.12. When using continuous BIQ scores (Table 2), AB did not correlate with total BIQ scores, *r*(67) = 0.09, *p* = .45, or with total SCARED scores, *r*(60) = −0.19, *p* = .15. As expected, total BIQ scores positively correlated with total SCARED scores, *r*(40) = 0.53, *p* < .001, with BI children scoring higher on anxiety symptoms than non-BI children, *t*(58) = −3.66, *p* = .001, *d* = −0.95 (Table 1). A lack of association between behavioral measures and BIQ scores or anxiety levels is common in the literature (e.g., Pérez-Edgar et al., 2010, Pérez-Edgar et al., 2011; Shechner et al., 2012), likely reflecting the low reliability of attention bias measured as a behavioral difference score using the dot-probe task (Van Bockstaele et al., 2014). In contrast, electrophysiological and imaging measures of bias derived from the task appear to show greater sensitivity and reliability (Britton et al., 2012; White et al., 2017b).

**Table 2:** Bivariate correlations for study variables.

Variable	1	2	3
1. BIQ scores			
2. AB	0.09		
3. Anxiety symptoms	0.53⁎	−0.19	

BIQ = Behavioral Inhibition Questionnaire. AB = attention bias.

⁎ *p* *<* .01.

Thirty-one percent of BI and 6.5% of non-BI children met the cutoff criteria (a score ≥ 8 on parent report) for SAD on the SCARED. When using primary caregiver's interview on the C-DISC, only two behaviorally inhibited children met diagnostic criteria for SAD while one non-inhibited children met criteria for GAD. Thus, this is a sample of generally healthy children with elevated symptoms, reflecting their risk status for developing an anxiety disorder.

### 3.2 fMRI Results

#### 3.2.1 Whole sample: faces > baseline

**Fig. 2:**
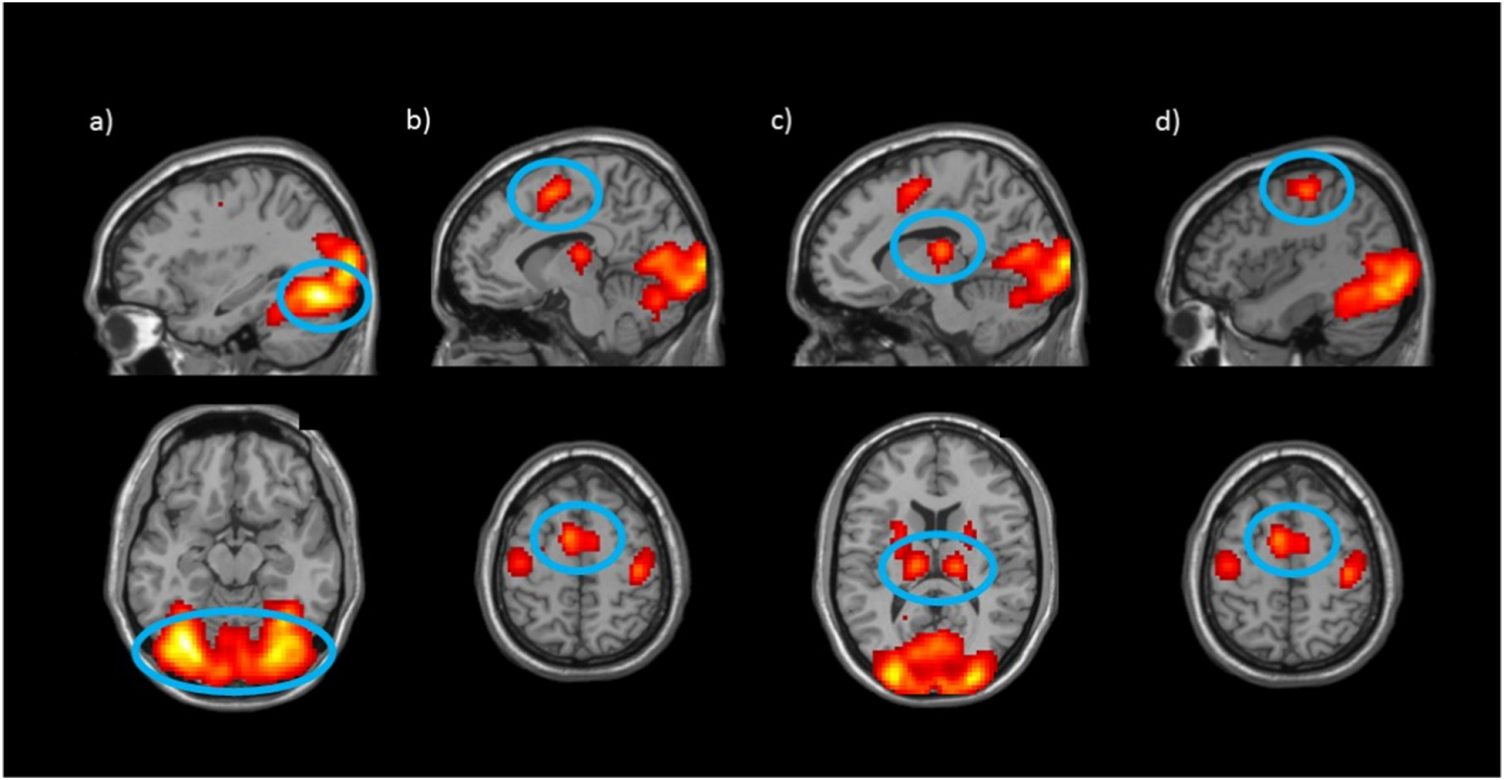
Whole sample Face > Baseline. The whole-brain analysis comparing presentation of face stimuli to baseline revealed activation significantly greater than zero across all participants in six regions: a) Fusiform, b) Supplementary Motor Area, c) Thalamus, and d) Precentral. Activation presented at *p* = .005 with a cluster extent threshold of 86 voxels.

A whole-brain one-sample *t*-test was carried out to characterize the task-related neural response (to face stimuli compared to baseline). This *t*-test identified 6 significant clusters in a contrast of faces (all task stimuli, both neutral-neutral and threat-neutral) relative to baseline trials (all other trial-frames that did not include faces) (Table 3). As expected, there was bilateral activation in Fusiform (a region involved in face processing; e.g., Grill-Spector et al., 2004; Kanwisher et al., 1997), bilateral Supplementary Motor Area, bilateral Thalamus, and Precentral gyri (Fig. 2).

**Table 3:** Activation clusters in response to faces relative to baseline that survived the *p* < .05 cluster-corrected thresholds at voxel-wise threshold of *p* = .005 identified in the one-sample *t*-test, for whole-brain (Fig. 2) analyses.

Region	Peak MNI coordinates			Cluster size (voxels)	*t*(66)
	x	y	z		
Whole-brain analysis					
Fusiform (B)	−33	−73	−14	7095	15.72
Supplementary Motor Area (B)	−9	2	55	296	9.74
Thalamus (L)	−12	−19	13	414	9.68
Thalamus (R)	15	−19	13	299	5.51
Precentral (L)	−42	−16	55	151	4.49
Precentral (R)	42	−22	55	156	4.47

R = right; L = left; B = bilateral.

#### 3.2.2 Whole sample: threat > neutral amygdala SVC

**Fig. 3:**
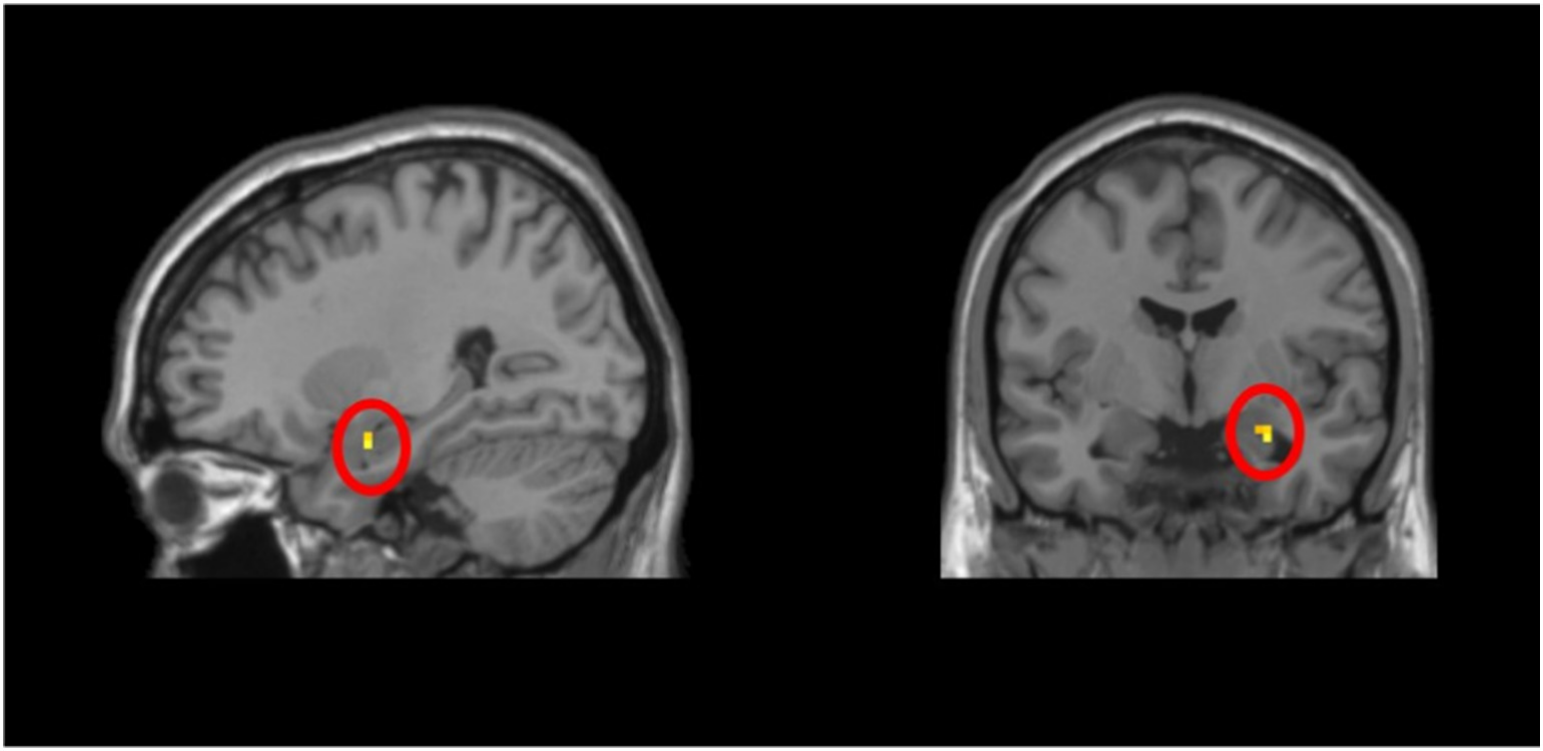
Whole sample Threat > Neutral amygdala small-volume correction (SVC). In response to Threat versus Neutral trials, activation in a priori anatomically-defined ROIs of right amygdala was significantly greater than zero across all children. Activation presented at *p* = .005 with cluster corrected to 3 voxels.

The one-sample *t*-test did not find significant activation (*p* < .005) in either amygdalae in response to threat versus neutral trials. However, the prior 3dClustSim version (i.e., prior to May 2015) indicated significant activation (*p* < .005) in the left amygdala ROI for the sample as a whole in response to threat versus neutral trials (Table 4; Fig. 3). The shift is likely due to the more conservative approach of the new model which requires 13 voxels for significance, versus 3 voxels in the prior approach.

**Table 4:** Activation clusters in response to threat relative to neutral trials that survived the *p* < .05 cluster-corrected thresholds at voxel-wise threshold of *p* = .005 identified in: (a) the one-sample *t*-test, for amygdala ROI (Fig. 3) and (b) the two-sample *t*-test, for BN > BI whole-brain analyses (Fig. 4).

Region	Peak MNI coordinates			Cluster size (voxels)	*t*(65)
	x	y	z		
*Whole Sample*, *ROI analysis; NT* *>* *NN*					
Amygdala (R)	27	−4	−20	3	3.30

*Two-Sample*, *Whole-brain analysis; NT* *>* *NN*, *BN* *>* *BI*					
Middle Frontal Gyri (B)	33	32	37	823	4.35
Inferior Frontal Triangularis (R)	30	17	25		
Middle Singulum (R)	12	14	31		
Postcentral Gyrus (L)	−54	−19	16	332	3.83
Putamen (L)	−27	−10	16		
Thalamus (L)	−6	−7	16		
Superior Temporal Gyrus (R)	51	−25	13	169	3.94
Insula (R)	27	−19	13		
Rolandic Operculum	39	−25	19		
Inferior Frontal Triangularis (L)	−42	17	22	166	4.28
Precentral Gyrus (L)	−24	−22	67	137	3.97
Middle Frontal Gyrus (R)	33	56	16	101	3.67

ROI = region of interest; R = right; L = left; B = bilateral.

#### 3.2.3 BI-group differences: threat > neutral, Non-BI > BI

**Fig. 4:**
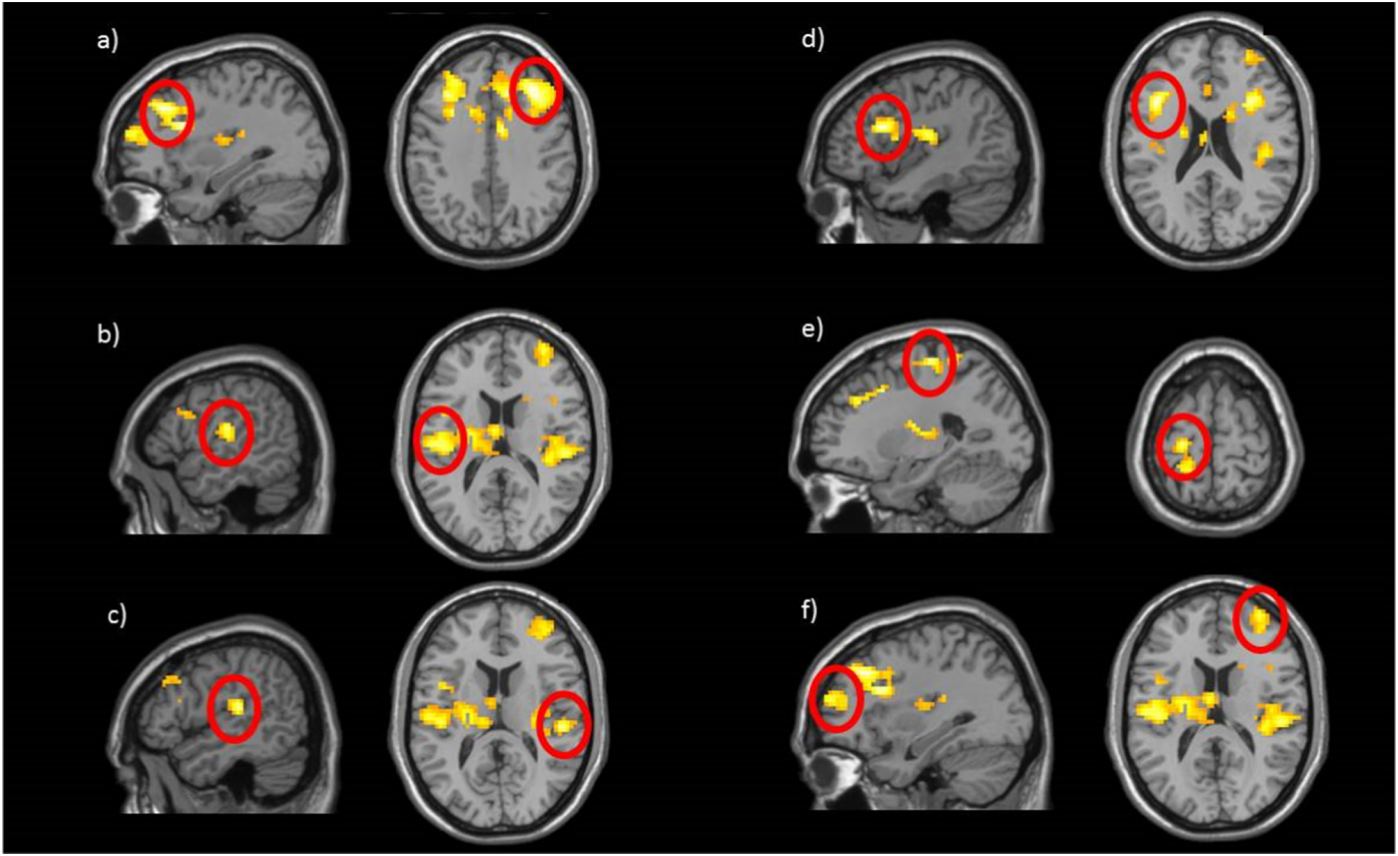
BI-group comparison on Threat > Neutral contrast: BN > BI. Compared to behaviorally inhibited (BI) children, behaviorally non-inhibited children (BN) showed greater activation in response to threat relative to neutral stimuli in six clusters: a) right Middle Frontal gyrus, b) left Postcentral gyrus, c) right Superior Temporal gyrus, d) left Inferior Frontal Triangularis, e) left Precentral gyrus, and f) right Middle Frontal Gyrus. Activation presented at *p* = .005 with a cluster extent threshold of 86 voxels.

This whole-brain two-sample *t*-test identified 6 significant clusters in a contrast of the non-BI group versus the BI group (Table 4; Fig. 4): bilateral Middle Frontal gyri, left Postcentral gyrus (encompassing left putamen and left thalamus), left Precentral gyrus and Inferior Frontal Triangularis, and right Superior Temporal gyrus (encompassing the right Insula).

#### 3.2.4 BI-group differences: threat > neutral, incongruent > congruent, BI > non-BI

**Fig. 5:**
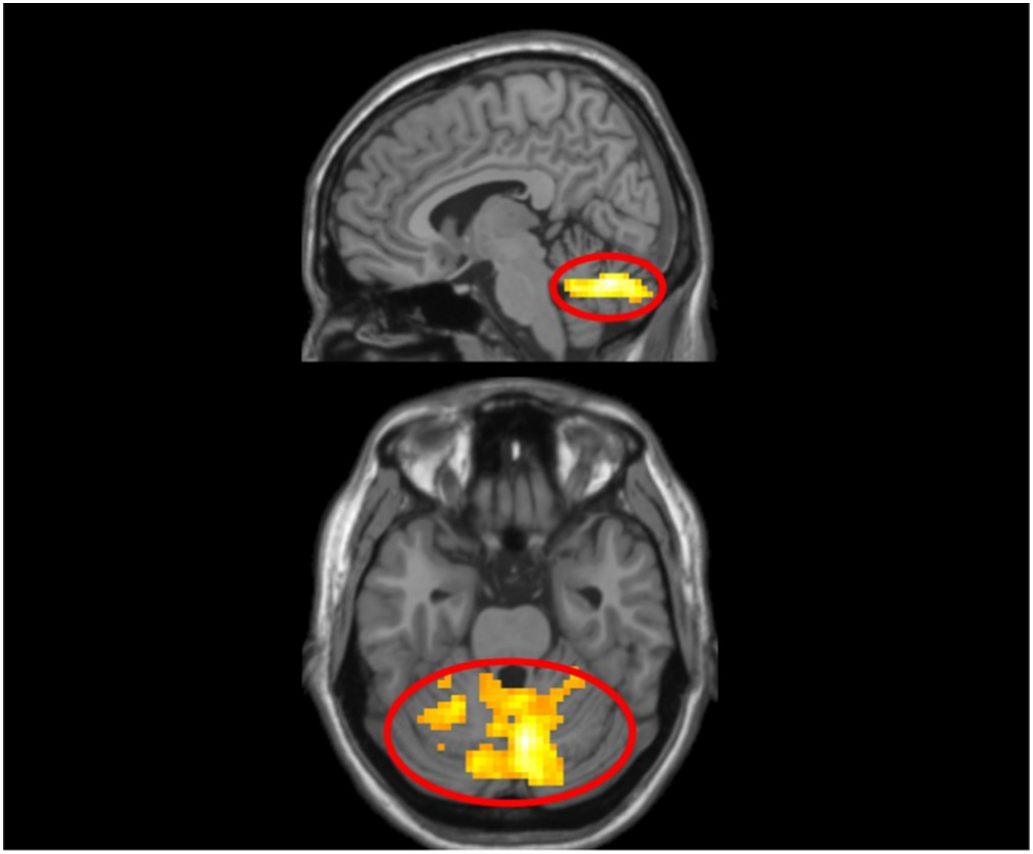
BI-group comparison on Incongruent > Congruent in Threat > Neutral contrast. Behaviorally inhibited children (BI) showed greater activation in one, bilateral Cerebellum cluster (peak MNI coordinates: 6, −67, −26 voxels) than non-BI children (BN) in response to threat relative to neutral stimuli. Activation presented at *p* = .005 with a cluster extent threshold of 86 voxels.

This whole-brain two-sample *t*-test identified 1 significant cluster in the bilateral cerebellum for the contrast of BI individuals versus non-BI individuals (Table 5; Fig. 5).

**Table 5:** BI-group comparison on Incongruent>Congruent in threat relative to neutral (NT > NN) trials that survived the *p* < .05 cluster-corrected thresholds at voxel-wise threshold of *p* = .005 identified in the whole-brain one-sample *t*-test for BI > BN analysis (Fig. 5).

Region	Peak MNI coordinates			Cluster size (voxels)	*t*(65)
	x	y	z		
*Incongruent* *>* *Congruent; BI* *>* *BN*					
Cerebellum Vermis7 (R)	6	−67	−26	822	4.42

ROI = region of interest; IFG = inferior frontal gyrus; R = right; L = left.

## 4 Discussion

This is the first study to investigate behavioral and neural responses to briefly displayed masked angry faces in behaviorally inhibited and non-inhibited children. The aim was to further clarify the pattern of neural response to socially-relevant threat in children at heightened temperamental risk for SAD, as previous studies had not focused on neural response to rapid, potentially pre-conscious stimuli. As expected, BI scores positively correlated with concurrent anxiety levels. However, neither BI nor anxiety scores correlated with behavioral AB. In response to the task, both temperament groups exhibited greater activation in the fusiform area, thalamus, supplementary motor area, and in prefrontal regions (precentral gyrus). Contrary to our expectation, our findings indicate that all children exhibited right amygdala reactivity in response to briefly presented threatening stimuli. Differential patterns of brain activity were evident between behaviorally inhibited and non-inhibited children in non-limbic regions (i.e., temporal and prefrontal lobes) that are involved in modulating the response to threat stimuli.

Our findings of amygdala activation across the sample may indicate that amygdala hyper-reactivity to ambiguous salient stimuli may help individuals learn to identify significant emotional and social cues. Monk et al. (2008) reported that only clinically anxious adolescents show increased amygdala activation in response to subliminal threat, which they interpreted as a risk marker for anxiety severity. They also noted negative coupling with prefrontal activation in anxious adolescents. Our sample is both younger and less anxious than the Monk et al. (2008) study, which may account for the differences in data patterns. It may be that early reactive patterns to ambiguous or degraded stimuli may lead to abnormal information-processing schemas and contribute to the development of psychopathology if they are not modulated (e.g., “top-down” modulation) over time (Duval et al., 2015; Taylor and Whalen, 2015). It may be that some inhibited children (like their non-inhibited counterparts) develop effective modulating circuitry, such that they do not go on to exhibit anxiety even in light of a hyper-reactive limbic response to threat. This could be one possible reason why some studies report that adults with higher, non-pathological trait anxiety showed heightened amygdala activity in response to fearful faces, compared to non-anxious adults (e.g., Bertolino et al., 2005; Stein et al., 2007). Longitudinal studies, noting trajectories of neural responses to threat and anxiety levels, will be needed to fully explore this potential relation. Larger samples will also help probe the strength of the effect. Here, the relation appears to be somewhat fragile as variation in analysis approach impacted the significance of the relation.

Although stimuli were presented briefly to participants, we found evidence in non-inhibited children for the involvement of regions linked to higher-level stimuli processing. This group showed a pattern of activation in prefrontal (bilateral middle frontal gyrus, bilateral inferior frontal truangularis, and precentral gyrus) and temporal (superior temporal gyrus and postcentral gyrus, encompassing medial regions: the insula, putamen, and thalamus) lobes in response to threatening stimuli, compared to neutral stimuli. These data indicate that, for non-BI children, fronto-limbic circuitry is activated in response to briefly presented threat cues. This activation may help to down-regulate, or modulate, responses to potential threat—particularly when the stimulus is degraded and made difficult to classify. This circuitry may act as a gate-keeper mechanism that decides what is, and what is not, treated as a real threat. In behaviorally inhibited or clinically anxious youth, this circuitry may be ineffective or muted, possibly leading to elevated experiences of threat and anxiety symptoms. However, we cannot address this question in the current sample given the low levels of anxiety diagnoses in the study.

In contrast, behaviorally inhibited children, relative to non-inhibited children, displayed greater cerebellar activation when contrasting the incongruent and congruent conditions within the neutral-threat trials. There is increasing recognition that the cerebellum is a contributing factor to executive functions, attention, and emotion processing (Schmahmann and Caplan, 2006). Studies have reported structural and functional links between the cerebellum and regions involved in the perception and regulation of emotionally salient materials such as limbic regions (e.g., Annoni et al., 2003) and prefrontal regions (e.g., Middleton and Strick, 2001; Rolls, 2004). For example, individuals with cerebellar lesions show decreased bilateral amygdala activity in response to threat face-stimuli, as well as decreased activity in the vlPFC and dlPFC (Turner et al., 2007) indicating that the cerebellum might play a role in triggering or maintaining the threat processing circuitry. Turner et al. (2007) also reported that cerebellar lesions were associated with increased activation in vmPFC, posterior insula, and ACC, suggesting alternative nodes for threat processing in the absence of cerebellum-amygdala activation.

Our findings in the incongruent-versus-congruent condition may suggest that individuals at-risk for anxiety activate the cerebellum to engage in monitoring and regulation of threat reactivity via allocation of attention to support performance, following the initial limbic response. Baumann and Mattingley (2012) reported that cerebellar regions topographically similar to the current study's findings were involved in reaction to primary emotion evoking stimuli (including anger and fear) during a prolonged stimuli presentation time of 4000 ms. This could indicate that cerebellar reactivity to threat (and other emotional stimuli) is protracted or extends past the initial stages of emotional processing. In this way, it may be a possible contributor to the fronto-limbic perturbations evident in inhibited children and adults in the larger literature (Fox et al., 2008). Further investigation is required to better characterize the function of the cerebellum in emotion processing, particularly its temporal effects on the fronto-limbic network.

There are several limitations to our study. First, in the process of analyzing fMRI data we excluded 15 BI and 8 non-BI participants (see Table 1) due to excess motion in the scanner. To an extent, this data loss disparity might reflect an expected anxiety-linked response to the scanner (e.g., elevated anxiety symptoms due to a loud and confined scanner environment; Eatough et al., 2009; Muehlhan et al., 2011). This loss pattern also reflects our cautious approach with analyzing our data as movement is a crucial marker of data integrity (Greene et al., 2016; Leroux et al., 2013; Raschle et al., 2012). A second limitation centers on our use of parent-reported BI rather than direct behavioral observation in toddlerhood. Scanning very young children while completing the dot-probe task is not feasible. The BIQ, as used here with strong selection cut-offs, is a reliable and valid approach to identifying behaviorally inhibited children (e.g., Coplan et al., 2009, Coplan et al., 2010). This approach allows researchers to examine the mechanisms associated with BI outside the rich, but relatively rare, context of a long-term, large-scale, longitudinal study. Finally, we do not know if the groups differed in their ability to perceive the masked stimuli, which could partially account for the observed activation patterns.

Even with these limitations, the current study points to the role of complex patterns of attention to threat in anxiety and risk for anxiety. Current models suggest that attention bias to threat may play a causal role in the emergence of anxiety (e.g., Mathews and MacLeod, 2002). Although studies experimentally manipulating attention bias have modulated levels of anxiety and stress reactivity in children and adults (Eldar et al., 2012; Eldar et al., 2008; MacLeod et al., 2002; Mathews and MacLeod, 2002), we know little regarding the underlying neural mechanisms. Recent work suggests that attention manipulations may increase frontal activation (vlPFC) while minimizing limbic (amygdala, insula) activation (Britton et al., 2015; Liu et al., 2018). However, this work has relied on extended (500 ms) presentation of threat stimuli. We do not know how, or if, attention bias modification impacts initial reactive responses to briefly presented stimuli. A more nuanced understanding of the time course of activation is needed as recent work has called into question the reliability and efficacy of treatments targeting attention and attention to threat (Rodebaugh et al., 2016).

The current study indicates that there are both distinct and overlapping patterns in how behaviorally inhibited and non-inhibited children process subliminal socially-relevant threat cues. We found that all children showed a (modest) activation response to masked threat faces in the amygdala, reflecting prior expectations for a limbic response to ambiguous threat. Non-BI children also displayed a distributed activation pattern relative to BI peers in the same contrast. Furthermore, behaviorally inhibited children showed heightened neural response in the cerebellum in trials that required shifting attention away from threat. The cerebellum is only recently gaining attention for influencing processes beyond motor control, extending to emotion processing. Further examination is required to help elucidate how distributed patterns of activity contribute to the processing of salient stimuli and threat into adolescence and early adulthood, and how these activation patterns come into play in temperamentally inhibited versus anxious individuals.
